# Solid electrolyte interphase formation between the Li_0.29_La_0.57_TiO_3_ solid-state electrolyte and a Li-metal anode: an *ab initio* molecular dynamics study

**DOI:** 10.1039/c9ra10984f

**Published:** 2020-03-02

**Authors:** Diego E. Galvez-Aranda, Jorge M. Seminario

**Affiliations:** Department of Chemical Engineering, Texas A&M University College Station TX 77843 USA seminario@tamu.edu; Department of Electrical and Computer Engineering, Texas A&M University College Station TX 77843 USA; Department of Materials Science and Engineering, Texas A&M University College Station TX 77843 USA

## Abstract

An *ab initio* molecular dynamics study of an electrochemical interface between a solid-state-electrolyte Li_0.29_La_0.57_TiO_3_ and Li-metal is performed to analyze interphase formation and evolution when external electric fields of 0, 0.5, 1.0 and 2.0 V Å^−1^ are applied. From this electrochemical stability analysis, it was concluded that lithium-oxide (Li_2_O) and lanthanum-oxide (La_2_O_3_) phases were formed at the electrolyte/anode interphase. As the electric field increased, oxygen from the electrolyte diffused through the Li-metal anode, increasing the amount of O from deeper crystallographic planes of the electrolyte that reacted with Li and La. A strong reduction of Ti was expected from their Bader charge variation from +3.5 in the bulk to +2.5 at the interface. Due to the loss of Li atoms from the anode to form Li-oxide at the interphase, vacancies were created on the Li-metal, causing anode structure amorphization near the Li-oxide phase and keeping the rest of the anode structure as BCC. Therefore, the interface was unstable because of the continuous Li-oxide and La-oxide formation and growth, which were more pronounced when increasing the external electric field.

## Abstract 2

An *ab initio* molecular dynamics study of an electrochemical interface between a solid-state-electrolyte Li_0.29_La_0.57_TiO_3_ and Li-metal to analyze interphase formation and evolution when external electric fields are applied.

## Graphical abstract

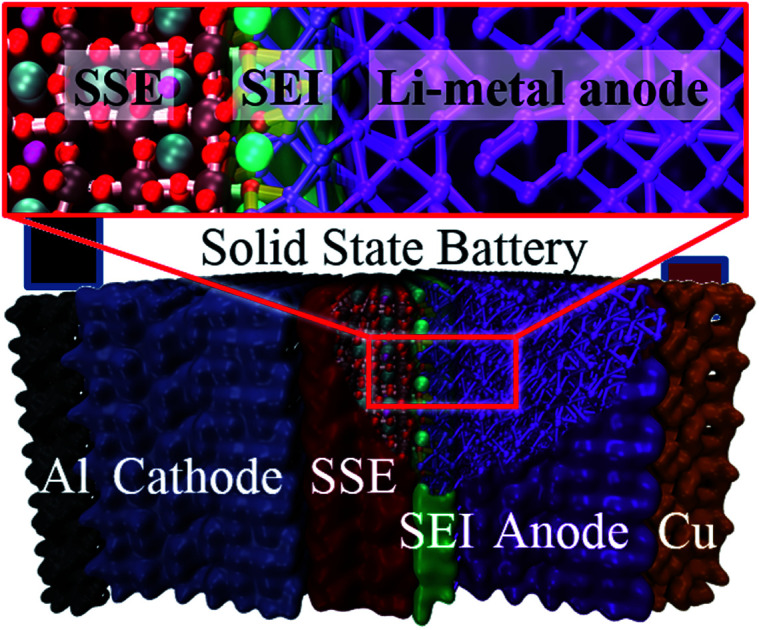

## 1. Introduction

Volume variations during lithiation of the Li-metal anode are among the main challenges in the development of new high energy density batteries;^1–4^ other phenomena such as solid electrolyte interphase formation^5–7^ on the lithium surface^8^ and lithium dendrite growth^9^ are also important to understand in order to achieve a stable Li-metal ion battery.

Solid state batteries (SSB)^10,11^ have been proposed as potential solutions to develop high energy density batteries, *i.e.*, the use of a solid-state electrolyte, instead of the traditional liquid electrolyte used in a lithium-ion battery (LIB).^12–15^ However, substituting only the electrolyte does not change the principle of operation of a SSB; still it is very similar to a LIB. Even though the principle of operation is the same for a SSB compared with a LIB, there are structural differences such as the no need of a separator in a SSB,^16^ which is needed with liquid electrolytes to prevent electronic short-circuits between electrodes.

Considering that the SSE/Li-metal interface is the most critical part of the SSB performance, one of the main challenges during fabrication of a SSB is the contact stability between SSE and the electrodes.^17^ For that reason, interfacial studies are a priority to determine if a material can be a potential SSE.^18^ The SSE must have a large electrochemical window and thermal stability at the interface with the lithium metal anode, assuring a controlled plating and stripping of lithium atoms in the anode. Therefore, computational tools such as density functional theory (DFT) calculations, classical molecular dynamics (CMD), and *ab initio* molecular dynamics (AIMD) allow us to study these interfaces in a detailed localized mode,^19^ including their morphology, composition, electrochemical, and thermal interfacial reactivity, complementing and expanding experimental information as some of these information cannot be obtained experimentally.

In this work, we study lithium lanthanum titanate perovskite ionic conductor (LLTO), Li_2/3−*x*_Li_3*x*_TiO_3,_ as SSE connected to a Li-metal anode. Li_2/3−*x*_Li_3*x*_TiO_3_ shows high ion mobility, reaching up to 10^−3^ S cm^−1^ at room temperature.^20^ In 1993, Inaguma *et al.*^21^ discovered that Li_0.34_La_0.51_TiO_2.94_ showed high ionic conductivity at room temperature, 2 × 10^−5^ S cm^−1^, then, several compositions of LLTO have been proposed as high ionic conductors and potential solid electrolytes materials.^22–25^ Several Li_2/3−*x*_Li_3*x*_TiO_3_ structures were tested, obtaining ionic conductivities from 10^−3^ to 10^−6^ S cm^−1^.^26,27^ Despite LLTO high conductivity, there are still some issues regarding the use of LLTO as a workable electrolyte. Reports indicate LLTO showed large grain boundaries (GB) resistance,^28,29^ which limits the Li transport. On the other hand, LLTO is not electrochemically stable in direct contact with Li-metal; a reduction of Ti from Ti^4+^ to Ti^3+^ takes place once the Li from the metal contact directly the Ti from the LLTO, increasing the electronic conductivity.^30–32^ The electronic conductivity of the mixed sample formed at the interface increases because Li from the metal reacts with the electrolyte, forming Li^+^, and the Li-ions diffuse into the vacancies sites in the LLTO electrolyte, forming metallic titanium.^26,33,34^

We focus this study on the Li_0.29_La_0.57_TiO_3_ solid electrolyte, which has been reported to have one of the highest ion conductivities at room temperature.^35^ We identify, at atomistic levels, issues leading to instabilities at the interface metal–SSE, reactions rates at the interface, identification of products and charge transfer. We apply an external electric field to emulate charging conditions and to study how the external electric field affects the stability of the electrochemical cell Li-metal/LLTO, showing how the external field affects the formation/degradation of the forming solid electrolyte interphase (SEI); thus, a better understanding of the phase formation can be obtained that in turn allows us to propose solutions to avoid or reduce undesired impacts of interfacial reactions.

## 2. Methodology

The Li-metal/Li_0.29_La_0.57_TiO_3_ cell consists of a slab of the Li_0.29_La_0.57_TiO_3_ deposited on top of a Li-metal surface. Because of the periodic boundary conditions, the system looks like a sandwich model, Li-metal/SSE/Li-metal. Two interfaces are analyzed, the Li-metal (001)/Li_0.29_La_0.57_TiO_3_ (002), which is a Li-metal (001) surface in contact with Li_0.29_La_0.57_TiO_3_ (002) composed of only O and La. The other interface is Li-metal (001)/Li_0.29_La_0.57_TiO_3_ (001), which is a Li metal (001) surface in contact with Li_0.29_La_0.57_TiO_3_ (001) surface composed of O and Ti atoms. The initial geometry of Li_0.29_La_0.57_TiO_3_ solid electrolyte slab is 14.2 Å with 8 layers (4Ti–O and 4La–Li layers) in the *z* direction. The first layer is the Li_0.29_La_0.57_TiO_3_ (001) plane, and the eight layer is the Li_0.29_La_0.57_TiO_3_ (002) plane. The electrolyte bulk is followed by 26 Å of Li metal material. The initial distance between the solid electrolyte and Li-metal bulk is 2.1 Å, chosen by previous energy convergence tests.

The interface is analyzed with the Born-approximation of AIMD^36^ simulations, in which the electronic Schrodinger equation is calculated using DFT^37^ within the projector augmented-wave approach (PAW) approach^38^ as coded in the Quantum Espresso program,^39^ with the Perdew–Burke–Ernzerhof (PBE) functional.^40^ A plane-wave energy cut-off of 40 Ry (*λ* = 0.5) and 27 *k*-points were used for the *k*-mesh. In addition, a cut-off of 200 Ry was used for the kinetic energy for charge density. This cut-off energy is larger than default values as we are working with slab model cells that include vacuum, requiring higher values of kinetic energy for charge density cut-off.^41,42^

Electronic degrees of freedom are relaxed at each AIMD time step (*τ*) of 1 fs. We performed temperature rescaling to keep the average temperature during the simulation at 300 K with a tolerance of ±20 K, following a Verlet algorithm to integrate the equations of motion for 20 ps. We analyze the density distribution, coordination polyhedra, charge transfer, and atomic diffusion at both interfaces, Li-metal (001)/Li_0.29_La_0.57_TiO_3_ (001) and Li-metal (001)/Li_0.29_La_0.57_TiO_3_ (002).

For the simulations under the effect of an external electric field a vacuum of 14 Å is added in the Li-metal/Li_0.29_La_0.57_TiO_3_ (001) electrochemical cell. The effect of an external electric field in the AIMD simulations is implemented with a saw-tooth potential added to the bare ionic potential of the interface.^43^ Three external electric fields are tested: *ε* = 0.5, 1.0 and 2.0 V Å^−1^. We compared the results when the external electric field is applied with the case when no-electric field is applied (0 V Å^−1^).

Lithium atoms originally placed in the solid electrolyte are referred as Li_se_ and lithium atoms originally placed in the metal anode are referred as Li_s_.

## 3. Results and discussion

**Fig. 1:**
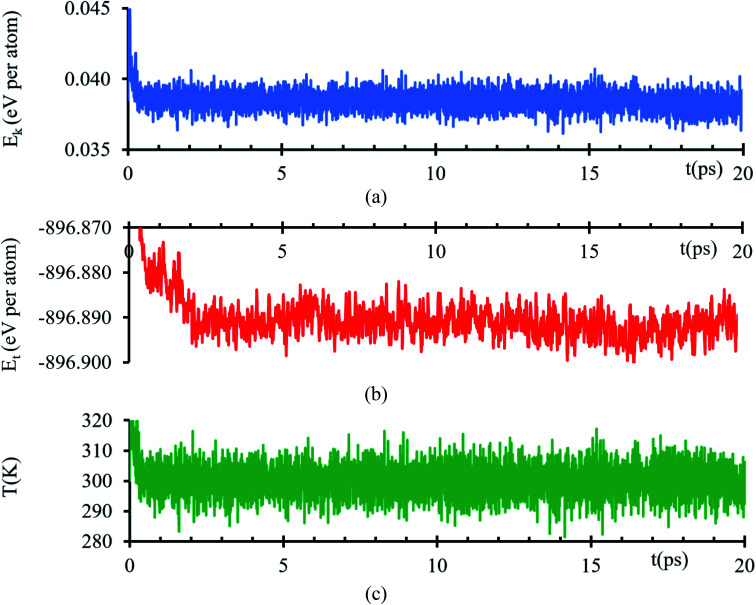
(a) Kinetic energy, (b) total energy and (c) temperature *versus* time of the AIMD simulation of the interfacial model.

Kinetic energy shows an initial transient decay in the first 500 ns (Fig. 1a), suggesting the time of early electronic rearrangements at the interface due to the encounter of the two surfaces, followed by a steady state behavior with small oscillations of 38 ± 1 meV per atom (Fig. 1a) compatible with thermal noise (∼27 meV at 300 K). A corresponding longer duration behavior is experienced in the total energy, a transient decay in the first 2.2 ps, suggesting early ionic rearrangements, followed by a steady energy of −896.892 ± 0.005 eV per atom (Fig. 1b) presenting no further considerable changes. The temperature is properly controlled; thus, the system always has a temperature of 300 K with a tolerance of ±15 K (Fig. 1c).

**Fig. 2:**
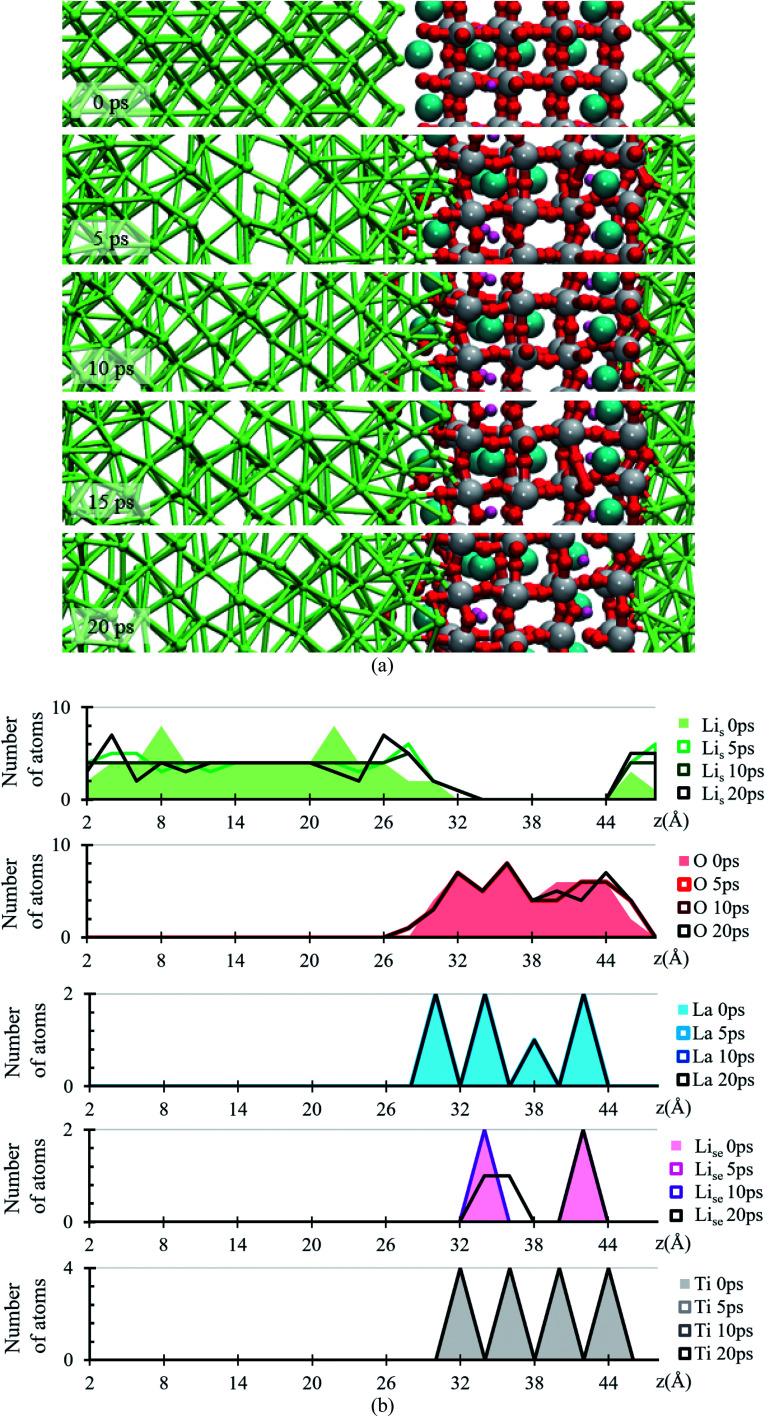
(a) Snapshots of the Li-metal/Li_0.29_La_0.57_TiO_3_ cell from 0 to 20 ps. (b) Atomic profiles of the number of atoms at 0, 5, 10, 15 and 20 ps along the *z*-axis of the Li-metal/Li_0.29_La_0.57_TiO_3_ cell. Li_s_ (green), Ti (gray), La (cyan), Li_se_ (pink), O (red).

During the evolution of the two interfaces, Li_0.29_La_0.57_TiO_3_ (001) and Li_0.29_La_0.57_TiO_3_ (002), in contact with Li-metal, when no electric field is applied for 20 ps, decomposition of the O atoms originally bonded to Ti occurs in the Li_0.29_La_0.57_TiO_3_ (002), causing the deformation of the polyhedral Ti–O from the internal layers of the solid electrolyte. Oxygen atoms form new bonds with Li-metal atoms. During the 20 ps of the simulation without electric field, Li–O interactions only occur at the interface level. Several internal layers of Li-metal keep their BCC structure along the simulation; however, reactions may continue after the 20 ps because the slow diffusion of O through the Li-metal generates Li–O as soon as O approaches Li atoms from the Li-metal. The formation of the Li–O phase favors the movement of La towards the Li-metal (Fig. 2a).

The atomic profile along the *z*-axis shows that the position of Ti and La atoms do not undergo major geometrical changes during the 20 ps of simulation, thus the crystallinity of the structure is not lost as those atoms only vibrate. O atoms originally placed at the Li_0.29_La_0.57_TiO_3_ (002)/anode interface migrate as opposed to those O at the Li_0.29_La_0.57_TiO_3_ (001)/anode interface that stay put after the optimization, without major changes in their positions near the interface. The presence of La at interface Li_0.29_La_0.57_TiO_3_ (001)/anode favors the diffusion of O towards the Li-metal anode. A rearrangement on the Li atoms from the metal anode during the simulation suggests that some of these Li could have reacted with the O atoms at the interface. Therefore, an analysis involving Bader charges calculation is performed to identify possible product formation at the interfaces (Fig. 2b).

**Fig. 3:**
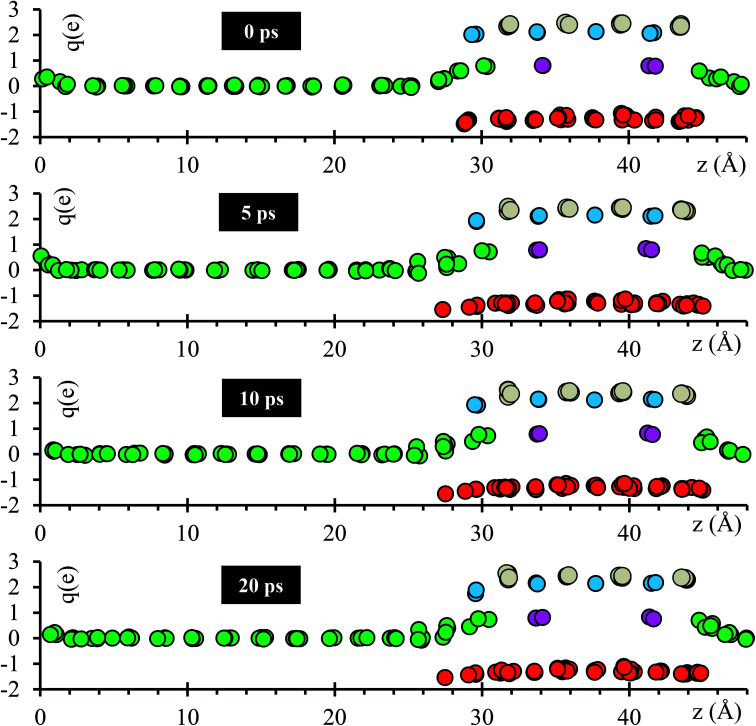
Time evolution of the atomic Bader charges at 0, 5, 10 and 20 ps. Li_se_ (violet), O (red), Li_s_ (green), La (cyan) and Ti (grey).

Atomic Bader charges are calculated to analyze time-dependent products formation at the interface. Li-ions in the SSE (Li_se_) feature charges of +1. Li from the metal anode (Li_s_) shows a net charge of 0; however, Li_s_ increases its charge from 0 to +1 at both interfaces as they get closer to the SSE. A slight gain in negative charge in the oxygen atoms at both interfaces is observed, from −1.3 of the oxygens inside the SSE to −1.5 of the oxygen at the interfaces. Ti atoms are already reduced, showing charges of ∼+3 in comparison with those of ∼+4 in the bulk. This reduction was reported experimentally once the LLTO SSE were in contact with the Li metal.^30–32^ The charge of Ti remains as +3 during the 20 ps of the simulation. La–O bond length is around ∼ 2.72 Å, which corresponds to the sum of the ionic radii, La^3+^ (1.36 Å) and O^2−^ (1.35 Å);^44^ however, in the Bader charge analysis we get +2 for La and −1.5 for O. The charge difference is distributed in the partially oxidized (positive) Li_s_ at both interfaces (Fig. 3).

**Fig. 4:**
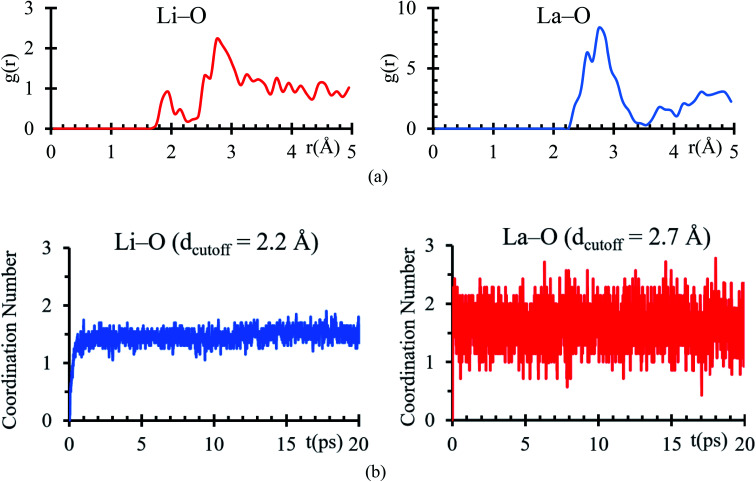
(a) RDF analysis of Li–O and La–O pair during the last 5 ps of the AIMD simulation. (b) Number of O atoms coordinated with (a) Li using a *d*_cutoff_ ≤ 2.2 Å and with (b) La using a *d*_cutoff_ ≤ 2.75 Å.

Bader charges of the La, Li and O atomic species suggest a possible formation of a solid electrolyte interface involving these atoms. An RDF analysis shows the formation of bonds Li–O with a clear peak at 1.95 Å, which is very close to 2.11 Å (ref. 44) (Fig. 4a), corresponding to the sum of ionic radii of O^2−^ (1.35 Å) and Li^1+^ (0.76 Å). For La–O pairs, the peak on the RDF analysis is at 2.75 Å, which is very near to 2.71 Å (ref. 44) corresponding to the sum of ionic radii of La^3+^ (1.36 Å) and O^2−^ (1.35 Å).

From the average number of Li around O atoms with a distance of less or equal to 2.2 Å, we find out that ∼2Li atoms are bonded to each O atom at the interface, coinciding with the number of Li on the neighborhood around every O atom in a Li_2_O crystal (Fig. 4b). To calculate the average number of O around La atoms, we used a distance smaller or equal than 2. 75 Å, finding an average of less than 2Li bonded to each La at the interface, which coincides with the number of Li on the neighborhood around every La in a La_2_O_3_ crystal (Fig. 4b).

**Fig. 5:**
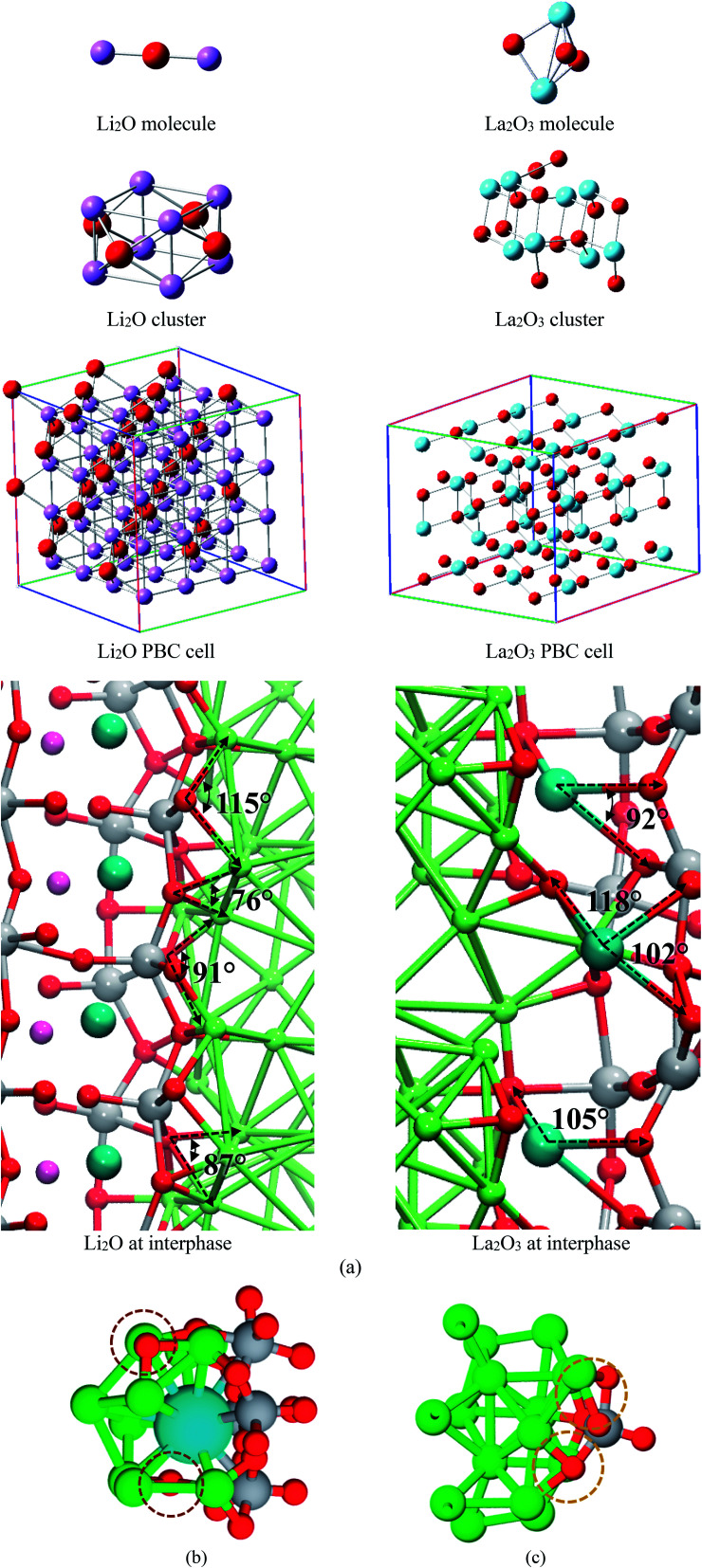
(a) Calculated lithium-oxide and lanthanum-oxide structures. (b) Structures found at both interfaces Li_0.29_La_0.57_TiO_3_ (001) and Li_0.29_La_0.57_TiO_3_ (002) in contact with Li metal. (a) Li–O–Ti caging a La atom due to rupture of some Ti–O bonds at interface. (c) Ti–O bonds remains unbroken when face the Li-metal.

We compare structural properties such as bond lengths and angles of the formed Li–O and La–O phases occurring at the interphase of the electrochemical cell with those from a lithium-oxide and lanthanum-oxide molecule, crystallographic cells optimized using DFT, and experimental data (Fig. 5a). We use calculated averages, except for the Li_2_O and La_2_O_3_ molecules. Li–O and La–O bonds lengths are similar in all the optimized cases of crystallographic structures (Table 1), angles Li–O–Li and O–La–O also shows values in good agreement among different structures; differences are found with the molecules but Li-oxide and La-oxide phases are not made of isolated molecules; they reassemble to a crystal structure (Table 1).

**Table 1:** Average lithium-oxide and lanthanum-oxide bond lengths (Å) and angles (°) from theory and experiment

Material	Parameter	Molecule	Crystal cluster	Crystal cell	Interphase	Experiment
Li_2_O	Li–O	1.62	1.83	1.99	2.1	1.96–2.15 (ref. 45 and 46)
	Li–O–Li	180.0	80.3	70.5	86.5	
La_2_O_3_	La–O	2.15	2.35	2.38	2.42	2.37 (ref. 47)
	La–O–La	77.2	110.5	112.2	106.3	

For the interfacial AIMD structure, the charge difference with the crystal cluster, crystal cell, and molecule depend on the environment. Notice that other atomic species, *e.g.*, Ti and Li, surrounds the Li_2_O and La_2_O_3_ formation at the interface where O and Li are in chemically bonded to La. Thus, adapting a variety of charges; however, almost all structures feature similar charges for O as well as for Li species. For La_2_O_3_, the formal charges are La(+3) and O(−2), and the corresponding calculated Bader charges have the same ratio as the calculated for the crystal cluster, cell, and the formal charges (Table 2). From these comparisons, we conclude that the new Li-oxide and La-oxide phases being formed at the interface are consistent with the Li_2_O and La_2_O_3_ crystallographic structure.

**Table 2:** Comparison of average Bader charges from typical lithium-oxide structures

System		Charge type			
		NBO		Bader	
		Molecule	Crystal cluster	Crystal cell	Interface
Li_2_O	Li	0.73	0.66	0.62	0.52
	O	−1.46	−1.33	−1.24	−1.40
La_2_O_3_	La	2.07	2.72	2.95	1.95
	O	−1.38	−1.91	−1.96	−1.32

Oxygen diffuses faster at the Li_0.29_La_0.57_TiO_3_ (002)/anode interface than at the Li_0.29_La_0.57_TiO_3_ (002)/anode interface. The presence of La at the Li_0.29_La_0.57_TiO_3_ (002)/anode interface helps a faster dissociation of O from the crystal structure originally bonded to Ti, allowing these O to make new bonds with the Li from the metal (Fig. 5b). Ti–O bond breaking does not occur at the Li_0.29_La_0.57_TiO_3_ (001)/anode interface, O forms bond with the Li from the metal anode, but these O remains bonded to the Ti (Fig. 5c).

**Fig. 6:**
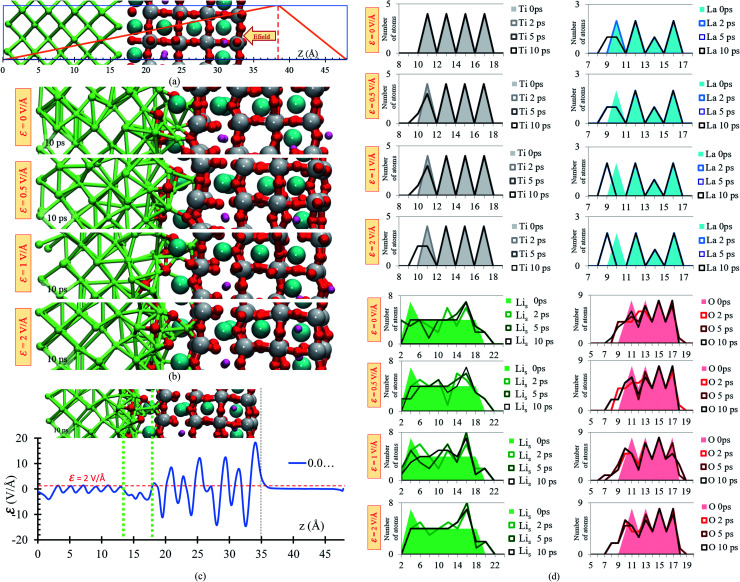
(a) Li_0.29_La_0.57_TiO_3_/Li-metal electrochemical cell under the application of an external electric field. (b) Structural changes of the Li_0.29_La_0.57_TiO_3_/Li-metal interface at 10 ps of applying external electric fields of *ε* = 0, 0.5, 1 and 2 V Å^−1^. (c) Profile average of the internal electric field component in the drift direction of transport of ions. (d) Atomic profile along the *z* axis at different time rates, for the four cases, *ε* = 0, 0.5, 1, 2 V Å^−1^.

A slab geometry is adopted to study interfacial behavior of the Li_0.29_La_0.57_TiO_3_/Li-metal under the effect of an external electric field. The slab of Li_0.29_La_0.57_TiO_3_/Li-metal interface has a size of 7.45 × 7.45 × 48 Å^3^. A saw-like potential bias is used. The direct bias part simulates an externally applied electric field. It is simply included to keep the periodicity of the potential, avoiding its growth from cell to cell. Therefore, the reverse bias region if left empty as any interaction in that region would correspond to unphysical effect not related to the system under study (Fig. 6a).

We perform *ab initio* molecular dynamics calculations under the effect of three electric field values, *ε* = 0, 0.5, 1, 2 V Å^−1^. We study how the interface evolves during 10 ps for all the applied fields, and identify the responses. The direction of the external electric field is parallel to the longitudinal axis (*z*-direction), from the electrolyte to the metal anode. In the four cases, *ε* = 0, 0.5, 1 and 2 V Å^−1^, a new phase is formed at the interface metal–SSE (Fig. 6b). These justify the use of the electric field of values of *ε* = 0, 0.5, 1, and 2 V Å^−1^ as they cover the range of values needed to properly polarize the electrolyte against the internal atomistic electric fields (Fig. 6c). The profile average of the internal electric field component in the drift direction of transport of ions reaches more than 10 V Å^−1^ in the solid electrolyte region; however, at the interface (between the green dotted lines) all the values are under 2 V Å^−1^. Therefore, that determines the upper limit of the range of fields needed to cross over and to analyze reactions on the interface. However, the electric field for a practical condition, *i.e.*, in a real Li-ion battery, is much smaller, ∼10^−6^ V Å^−1^, but it may change a few orders of magnitude up or down according to the specific characteristics of the battery such as anode–cathode average distance, rate of charge, type of charging, conductivity of components, geometry of the cell, among several others.

Structural changes are analyzed calculating the atomic profiles along the direction of the electric field (*z*-direction), thus we can obtain more insights regarding interfacial structural changes. Atomic profiles are calculated at 0, 2, 5 and 10 ps for the Li_0.29_La_0.57_TiO_3_/Li-metal cell. Atomic species (O, La, Ti) migrated from the solid electrolyte towards the Li-metal due to the application of the external electric field. Oxygen atoms originally belonging to the solid electrolyte are the first atomic specie from the solid electrolyte that reacts in contact with the Li-metal. The migration of O to the metal anode increases as the applying external electric field increases. Shorter displacements are observed for the heaviest atoms in the electrolyte, Ti and La, moving towards the Li-metal. The migration of Ti and La to the metal anode increases as the applying external electric field increases. Likewise, some Li that are part of the Li-metal (Li_s_) diffuse towards the SSE. Based on the atomic profiles, the forming interface is composed in its majority of O and Li_s_ atoms during the first 10 ps, and as the electric field increases, the formation of a new interface is favored.

Overall, structural changes occurring at the interface due to SSE decomposition through the cell shows a stability when no electric field is applied, but the reaction rates increase at the interface when electric fields are applied. At 10 ps, 19 bonds Li–O are formed at the interphase when no electric field (0 V Å^−1^) is applied and 26 bonds Li–O are formed at the interphase when 2 V Å^−1^ is applied; in both cases, the distance cutoff to define a Li–O bond length is 2.2 Å, as explained earlier. Reactions involving La and Ti occur in smaller rates because their slower motion towards the metal anode, six La–O bonds are formed at the interphase when no field is applied and eight when 2 V Å^−1^ is applied. In both cases the cutoff distance to define a La–O bond is 2.75 Å as described above. However, the interface is constantly changing, and the reaction rates could change too. For example, as more oxygens react with Li, Ti and La are fully exposed to the metal anode, allowing new reactions as the formation of lanthanum-oxide phase taking place in addition to those involving only Li_s_ and O in the first 10 ps of simulation (Fig. 6c).

Using a Mean Square Displacement (MSD) analysis, we find out trends toward stability of the phase formation occurring at the interface metal–SSE. If the slope of the MSD curve is larger than 0, we expect that the atomic specie continues moving, thus further reactions are highly expected; however, if the slope tends to 0, the atomic specie has reached an stable position in which only vibrates, thus further reactions are less probable to take place. Considering that we are applying an external electric field, reactions involving atomic species that are stable during the first 10 ps might take place after. To identify those possible reactions, we studied the charge and bonds formation/breaking trends at times larger than 10 ps.

**Fig. 7:**
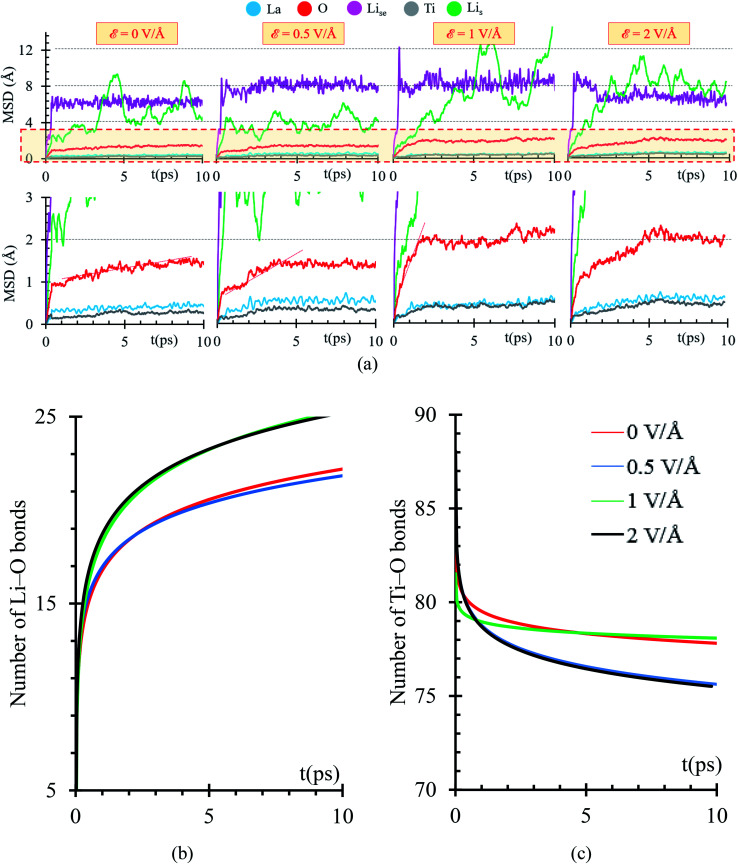
(a) MSD of Li_se_ (violet), O (red), Ti (gray), Li_s_ (green) and La (cyan), under *ε* = 0, 0.5, 1, and 2 V Å^−1^. Number of (b) Li–O and (c) Ti–O bonds under the application of external electric fields: *ε* = 0, 0.5, 1, and 2 V Å^−1^.

MSD curves show that the electric field slightly increases the diffusion of the heaviest atoms, Ti and La, suggesting that reactions might take place at times larger than 10 ps. Diffusion of Li-ions and Li_se_ feature a slight increase as the electric field increases, thus further reactions might take place as these atoms approach the interface with the metal anode. Li_s_ atoms from the metal anode do not follow a constant trend on their MSD curves because Li_s_ atoms are mostly neutral. They are not directly affected by the electric field; however, at the interphase, Li_s_ reacts with oxygen from the SSE forming a Li-oxide phase at the interface; therefore, vacancies on the metal anode are created, and a re-arrangement of the Li_s_ occurs, reflecting as peaks on the MSD curve or as diffusion of Li_s_ to fill up the created vacancies. O atoms show an initial slope larger than 0 in the MSD curve, occurring at the first picosecond due to initial reactions with Li_s_ at the interphase. The slope of the MSD curve during the first picoseconds increases as the applying external electric field increases. Then, the slope of the MSD curve decreases from 1 Å s^−1^ to a range between 0.05 to 0.11 Å s^−1^ depending on the value of the external electric field. As the simulation progresses further than 10 ps, MSD curves could change, as La and Ti atoms approach the interface and participate in new reactions at the interface. For *ε* = 0, further reactions involving O beyond 10 ps are expected because the slope of the MSD is larger than 0 (Fig. 7a).

Initial fast reactions occurring in the first picosecond of simulation in all the cases (*ε* = 0, 0.5, 1 and 2 V Å^−1^) form Li-oxide phase at the interface. As the electric field increases, it favors the formation of more Li–O. In all cases, the electric field drives the oxygen to diffuse through the Li metal. Reactions occur faster in the first picosecond of applying the external electric field. After the first picosecond, the Li-oxide phase is formed, but reactions are still occurring in slower rates than the ones occurring before the first picosecond. The rate of Li–O bond formation in the first picosecond is about 16 bonds per ps and in the following picosecond this value decreases up to 1 bond per ps. Initial reactions occur faster because oxygens from the Li_0.29_La_0.57_TiO_3_ (100) facet are near to the Li_s_ from the metal anode. Once, the initial Li-oxide phase is formed, Li–O bonds formation continues due to the effect of the electric field but at smaller rates because further oxygens arrive from deeper layers in the solid electrolyte.

Once at the interphase, oxygens reach Li_s_ from the metal, allowing the formation of a thicker Li-oxide phase (Fig. 7b). Simultaneously to the formation of Li–O, we observe decomposition of the Ti–O bonds; therefore, the oxygens reacting with the Li-metal are originally bonded to Ti. As the electric field increases, more O atoms can break their bonds with Ti (Fig. 7c).

**Fig. 8:**
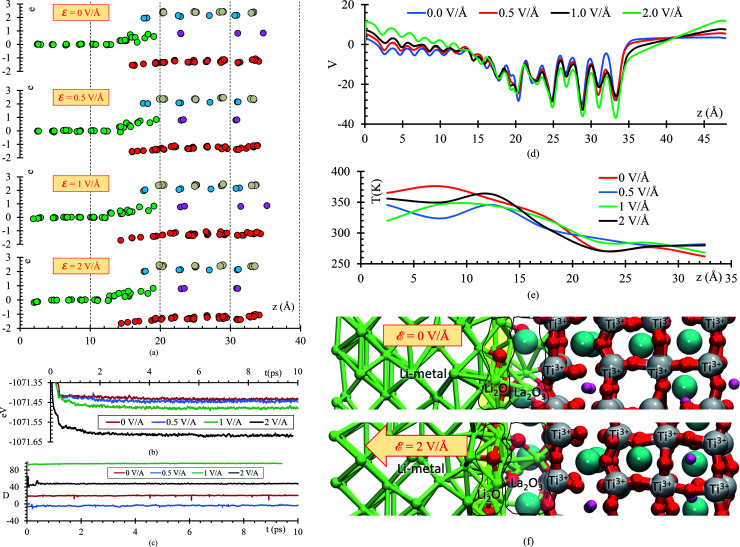
(a) Charges at 10 ps when *ε* = 0 V Å^−1^, *ε* = 0.5 V Å^−1^, *ε* = 1 V Å^−1^, and *ε* = 2 V Å^−1^. (b) Total energy, (c) dipole moment, (d) average potential energy, (e) temperature profiles along the direction of the electric field (*z*-axis) when external electric fields are applied, and (f) structures at 10 ps without and with the application of an external electric field of *ε* = 2 V Å^−1^.

To further analyze the behavior of the Li_0.29_La_0.57_TiO_3_/Li-metal interface, atomic Bader charges are calculated (Fig. 8a) for all the atomic species in the cell at 10 ps, for all *ε* = 0, 0.5, 1, and 2 V Å^−1^. In all the cases, we observe that Li_s_ gain charge throughout the 10 ps of the AIMD simulations. Li_s_ in the metal anode have a Bader charge of 0, but as Li_s_ approach the interface, Li_s_ reach an ionic behavior having a charge close to +0.8. Simultaneously, a decrease in the atomic charge of O is observed as oxygens diffuse through the metal anode and react with Li_s_. O in the SSE have an atomic charge of ∼−1.3, and at the interphase the O charge decreases up to ∼−1.6.

The stoichiometry of Li-oxide is Li_2_O with formal charges of +1 for Li, and −2 for O; therefore, we can clearly establish that the phase formed at the interface is a Li-oxide because the calculated average atomic charges of O and Li_s_ are −1.6 and +0.8, respectively.

They are also in agreement with the formal charges. Even though O atoms are originally bonded to the Ti, and Ti–O bonds are broken, we do not observe a clear change on the Ti Bader charges. The reduction of the Ti occurs at the very beginning of the simulation, having a Bader charge of +2.5 in comparison with the calculated Bader charge of +3.6 for the Ti in the bulk of the SSE. During the 10 ps of the simulation, we observe displacement of O through the Li-metal anode. The difference in number of broken Ti–O bonds is only 3 between *ε* = 0 and *ε* = 2 V Å^−1^. The overall displacement of O is about ∼2 Å along the longitudinal axis in the direction of the applied external electric field. Therefore, the neighborhood of the Ti when the Bader charge analysis is done, do not show much difference, obtaining similar charges of ∼2.5 along the 10 ps of the simulation for both cases, *ε* = 0 and *ε* = 2 V Å^−1^.

As the electric field increases, transport and reaction processes increase, thus further reactions that might occur later can be characterized without increasing the simulation time. Comparing the total energy curve for all 4 cases, *ε* = 0, 0.5, 1.0 and 2 V Å^−1^, we observe that the case of 2 V Å^−1^ features the lowest energy (Fig. 8b); therefore, the formation of Li-oxide and La-oxide phase and further reduction of Ti, take place because they are more stable compared with the case of 0 V Å^−1^, in which these reactions have not taking place yet, or are occurring at a much lower rate than at the 2 V Å^−1^ case. The dipole moment along the longitudinal direction, the direction of the electric field, is in average −4 D when no-electric field is applied. When an external electric field is applied, the dipole moment increases responding linearly to the value of the external electric field (Fig. 8c). The external electric field modifies the total potential along the longitudinal axis, for the atoms on the electrolyte we observe that the average potential decreases as the applying external electric field increases; however, for the Li-metal anode, the average potential increases as the applying external electric field increases (Fig. 8d). The temperature profile shows that the atoms in the Li-metal are in a higher temperature compared with the solid electrolyte, due to the reactions happening in the interphase, the Li, now ionized due to the formation of the SEI have greater kinetic energy that the atoms in the solid electrolyte. We observe this behavior in all four cases, with and without applying an external electric field (Fig. 8e). Finally, we identify two phases formed at the interface: Li_2_O and La_2_O_3_. The Li_2_O phase is formed closer to the Li-metal and the La_2_O_3_ is formed closer to the SSE (Fig. 8f).

## 4. Conclusions

Lithium-oxide and lanthanum-oxide phases are formed at the interphase Li-metal/La_0.43_Li_0.25_TiO_3_. Presence of La at the interphase helps to break the Ti–O bonds creating the Li–O bonds. Interfacial reactions La_0.43_Li_0.25_TiO_3_/Li-metal are mainly Li–O and La–O bond formation. O are taken from the TiO_3_ structure, breaking the original Ti–O bonds from the solid electrolyte; however, only a few O leave the solid electrolyte structure, mainly from the closest plane to the Li-metal anode. The lithium-oxide phase is formed faster than the lanthanum-oxide because oxygen atoms are in contact with the Li-metal anode. Once the lithium-oxide phase is formed, the La are exposed to the interface allowing the formation of a lanthanum-oxide phase.

From an MSD analysis, we conclude that the oxygen atoms migrate at a constant positive rate, demonstrating that these atoms are displacing towards the Li-metal anode; therefore, besides the initial and fast Li-oxide formation at the interface, further reactions are expected such as the formation of a lanthanum-oxide phase. The reduction of Ti occurs at the very beginning of the simulation, showing a Bader charge of +2.5 in comparison with +3.5 calculated in the SSE bulk. We have identified two phases formed at the interface: Li_2_O and La_2_O_3_. The Li_2_O phase is formed closer to the Li-metal and the La_2_O_3_ is formed closer to the SSE.

During the charging of a battery, an external electric field is applied, thus it is extremely important to analyze its effects on the new formed phases, lithium-oxide and lanthanum-oxide. The electric fields need to cover a range of values able to properly polarize the electrolyte against the internal atomistic electric fields, allowing as a result, the charge of the anode. Therefore, three external electric fields are applied, *ε* = 0.5, 1 and 2 V Å^−1^. Initial formation of lithium-oxide phase is observed for all cases, *ε* = 0, 0.5, 1 and 2 V Å^−1^; however, as *ε* increases, the formation of the lithium-oxide occurs faster and simultaneously; a faster decomposition of Ti–O bonds occur. As the field increases, O goes further inside the metal anode, reacting with more Li from the metal and accelerating the formation of the lanthanum-oxide phase. Notice that the electric field for a commercial Li-ion battery, is in the range of ∼10^−6^ V Å^−1^ but it may change a few orders of magnitude up or down according to the specific characteristics of the battery (anode–cathode average distance, rate of charge, type of charging, conductivity of components, geometry of the cell, among several others).

We identified initial reactions (Li-oxide and La-oxide formation) and established some mechanisms suggesting further reactions (Ti reduction) occurring at the Li_0.29_La_0.57_TiO_3_ (002)/Li-metal interface. The reaction rate at the interface increases as the applied external electric field increases, showing an electrochemical instability of the Li_0.29_La_0.57_TiO_3_ (002)/Li-metal interface. These results could be generalized to several other surfaces as well but not the rate of formation because each electrode surface possess distinct energetics. The products formed at the interface (La_2_O_3_ and Li_2_O) have been reported also experimentally, and in the experiments the contact surface is not only in one surface plane. On the other hand the metal expansion due to the arrival of Li-ions during the charging process and the depletion of them during discharge certainly affect the structure of the SEI causing its mechanical fracture and this expansion combined with the high reactivity of Li-metal with most of electrolytes may also lead to the formation of dendrites that eventually can short-circuit the battery. These are the major reasons why Li-metal batteries are not commercially used yet.

## Conflicts of interest

There are no conflicts to declare.

## Supplementary Material
